# The implausible “in vivo” role of hydrogen peroxide as an antimicrobial factor produced by vaginal microbiota

**DOI:** 10.1186/s40168-018-0418-3

**Published:** 2018-02-06

**Authors:** Gilda Tachedjian, Deirdre E. O’Hanlon, Jacques Ravel

**Affiliations:** 1 Disease Elimination Program, Life Sciences Discipline, Burnet Institute, 85 Commercial Rd, Melbourne, Victoria 3004 Australia; 2 Department of Microbiology, Monash University, Clayton, VIC 3168 Australia; 3 Department of Microbiology and Immunology, The University of Melbourne, The Peter Doherty Institute for Infection and Immunity, Melbourne, VIC 3010 Australia; 4 School of Science, College of Science, Engineering and Health, RMIT University, Melbourne, Victoria 3000 Australia; 5 Institute for Genome Sciences, University of Maryland School of Medicine, 801 West Baltimore Street, Baltimore, MD 21201 USA; 6 Department of Microbiology and Immunology, University of Maryland School of Medicine, Baltimore, MD 21201 USA

## Abstract

In the cervicovaginal environment, the production of hydrogen peroxide (H_2_O_2_) by vaginal *Lactobacillus* spp. is often mentioned as a critical factor to the in vivo vaginal microbiota antimicrobial properties. We present several lines of evidence that support the implausibility of H_2_O_2_ as an “in vivo” contributor to the cervicovaginal milieu antimicrobial properties. An alternative explanation is proposed, supported by previous reports ascribing protective and antimicrobial properties to other factors produced by *Lactobacillus* spp. capable of generating H_2_O_2_. Under this proposal, lactic acid rather than H_2_O_2_ plays an important role in the antimicrobial properties of protective vaginal *Lactobacillus* spp. We hope this commentary will help future research focus on more plausible mechanisms by which vaginal *Lactobacillus* spp. exert their antimicrobial and beneficial properties, and which have in vivo and translational relevance.

## Main text

In 1892, Albert Doderlein first described the presence of Gram-positive bacilli in the vagina of healthy reproductive-age women with low vaginal pH [1]. He considered the anti-staphylococci activities of the vaginal bacilli and the bactericidal action of the vaginal secretions to be due to the lactic acid produced by the bacilli [2]. His findings and those of others led to the proposal that vaginal acidification by lactic acid producing *Lactobacillus* spp. is the primary mechanism by which these bacteria contribute to the protection against reproductive tract pathogens [1, 2]. In addition to releasing organic acid metabolites (e.g., lactic acid) known to provide antimicrobial and immunomodulatory properties, *Lactobacillus* spp. can outcompete other bacteria at the epithelial mucosa as well as releasing bacteriocins, surfactants, antimicrobial proteins, or peptides [3–5]. In the 1990s, *Lactobacillus* species that produced H_2_O_2_ gained favour as being antimicrobial and synonymous with the presence of an optimal protective vaginal microbiota in reproductive-age women [6]. The protective role ascribed to H_2_O_2_ largely stems from epidemiological studies linking the presence in the vagina of H_2_O_2_-producing *Lactobacillus* spp. with a decreased risk for bacterial vaginosis (BV) [6–9], sexually transmitted infections [10, 11], as well as adverse birth outcomes [12], compared to women harboring non-H_2_O_2_-producing vaginal *Lactobacillus*. Unfortunately, these epidemiological observations were interpreted into the now widely accepted statement that “in vivo, *Lactobacillus* spp. exerts its antimicrobial properties through the production of H_2_O_2_”. While we do not dispute these epidemiological studies, the notion that in vivo H_2_O_2_ production by vaginal *Lactobacillus* spp. is an important factor contributing to the antimicrobial properties of these species is highly unlikely in the context of physiological conditions present in the lower female reproductive tract (FRT). In this commentary, several lines of evidence are presented that support the implausibility of “in vivo” H_2_O_2_ as an antimicrobial factor in the cervicovaginal environment. An alternative explanation is proposed, supported by previous reports ascribing protective and antimicrobial properties to other factors produced by *Lactobacillus* spp. capable of generating H_2_O_2_. Under this proposal, lactic acid rather than H_2_O_2_ plays an important role in the antimicrobial properties of protective vaginal *Lactobacillus* spp.

A fact that is not disputed is that in any microbial system, no matter the mechanism (pyruvate oxidase, lactate oxidase or NADH oxidase or NADH-dependent flavin mononucleotide reductase) [13], O_2_ is required to produce H_2_O_2_, and in large amounts to achieve H_2_O_2_ concentrations necessary to have antimicrobial activity in the vaginal environment [14–16]. Thus, a major question is how much oxygen is present to support this reaction in the vagina. The cervicovaginal environment is microaerobic (hypoxic) [17]. The mean cervicovaginal O_2_ levels range from 15 to 35 mmHg (2%), which is much lower than atmospheric levels of 160 mmHg (21%) [17–19]. Conversely, the cervicovaginal CO_2_ partial pressure ranges from 35 to 55 mmHg (or 5%), which is considerably higher than atmospheric levels (i.e., 5 mmHg, 0.04%) [17–19]. Only transient increases in O_2,_ that rarely achieve atmospheric levels, have been reported upon or after tampon or diaphragm insertion, sexual arousal [17, 18, 20, 21], and probably sexual intercourse. Vaginal *Lactobacillus* spp., like most *Lactobacillus* spp., are aerotolerant anaerobes and some do produce H_2_O_2_ when propagated under aerobic conditions, such as aeration by flask shaking [14, 15]. Conversely, lactic acid is predominately produced and at high concentrations under hypoxic conditions [19, 22]. Consistent with these in vitro observations and the low vaginal O_2_ levels measured in vivo, H_2_O_2_-producing vaginal *Lactobacillus* spp. have been shown to make little or no H_2_O_2_ in the context of the hypoxic cervicovaginal environment [16, 23].

The reported production of H_2_O_2_ by vaginal *Lactobacillus* spp. is measured using artificial in vitro conditions that do not recapitulate the hypoxic cervicovaginal environment [6, 14]. Consistent with this observation, the literature assigning an antimicrobial role for H_2_O_2_ describe experiments where *Lactobacillus* isolates are cultured under aerobic conditions to facilitate production and detection of H_2_O_2_ [6, 13–15, 24–26] (Table 1). Antimicrobial activity of H_2_O_2_ is observed in vitro in protein-free salt solutions, conditions that are not consistent with those in the cervicovaginal environment and an in vivo antimicrobial role for H_2_O_2_. Supporting this conclusion is the finding that H_2_O_2_ is inactivated by the reducing capabilities of cervicovaginal fluid (CVF) and semen [16, 23]. H_2_O_2_ measured in fully aerobic CVF is only 23 ± 5 μM [16]; however, CVF and semen completely reduce 1 mM and 10 mM added H_2_O_2_, respectively [16]. The addition of as little as 1% CVF supernatant completely abolishes the pathogen-inactivation by aerobically grown H_2_O_2_-producing *Lactobacillus* spp. [16] demonstrated by Klebanoff et al. [6]. In in vitro assays, physiological concentrations of H_2_O_2_ (< 100 μM) found in vaginal fluids, even when potentiated with myeloperoxidase, fail to inactivate bacterial vaginosis associated microbes, as well as bacterial (*Neisseria gonorrhoeae*) and viral (HSV-2) pathogens [16, 23]. Supra-physiological levels (10 mM) of H_2_O_2_ inactivate only one of 17 BV-associated bacterial species tested while completely inactivating four major vaginal *Lactobacillus* spp., *L. crispatus*, *L. gasseri*, *L. jensenii*, and *L. iners* [23]. These findings do not support an antimicrobial role for H_2_O_2_ in the cervicovaginal environment; however, the association between H_2_O_2_-producing strains of *Lactobacillus* spp. and favorable reproductive and urogenital outcomes may represent an in vitro marker for vaginal strains with beneficial properties. We put forth that other functions associated with these isolates capable of producing H_2_O_2_ in vitro must be responsible for their beneficial properties [3, 14, 24, 27]. In support of this proposal are issues with in vitro measurement of H_2_O_2_ production, which can be affected by growth kinetics. As such, a third of non-H_2_O_2_-producing strains were shown to ‘convert’ to H_2_O_2_ producers when the nutritional medium was reformulated [25] and in another study, 44% of non-H_2_O_2_-producing strains became producers when the time allowed for H_2_O_2_ production was increased from 30 to 60 min [12], vividly demonstrating that the difference is one of rate rather than absolute ability. Consistent with these findings, and the hypothesis that H_2_O_2_ is preferentially produced through NADH-dependent flavin mono-nucleotide reductase in lactic acid bacteria [13, 28, 29], an analysis of 125 *L. iners* genomes assembled from metagenomics datasets, showed that all genomes carry both genes found responsible for H_2_O_2_ production in *L. johnsonii* [13] and other members of the *L. acidophilus* group (data not shown). These finding severely weaken the association between production of H_2_O_2_ by vaginal *Lactobacillus* species and protection against BV, STI, and other adverse outcomes.

**Table 1:** Concentrations of hydrogen peroxide and lactic acid produced by *Lactobacillus* spp. under different conditions and concentrations necessary to inactivate HIV and BV-associated bacteria

Conditions	H_2_O_2_ [mM]		Lactic acid [mM]	
Culture medium under hypoxic conditions	Undetectable	[46]	160–250	[22, 47]
Aerated culture medium	3.34–4.39	[46]	45	[22]
*Lactobacillus*-dominated CVF under hypoxic conditions	Undetectable	[16]	110	[23]
Aerated *Lactobacillus*-dominated CVF	0.023	[16]	63	[23]
Inactivation of HIV in culture medium	5	[48]	33	[32]
Inactivation of BV-associated bacteria in culture medium	10	[23]	55–110	[23]
Inactivation of HIV in the presence of CVF	Undetermined		33–110	[32]
Inactivation of BV-associated bacteria in the presence of CVF	> 1000	[23]	55–110	[23]
Inactivation of *Lactobacillus* in culture medium	> 1000	[23]	> 1000	[23]

Vaginal *Lactobacillus* spp. produces lactic acid through the fermentation of polysaccharides, including glucose, under hypoxic conditions [30, 31]. In women of reproductive-age, harboring *Lactobacillus*-dominated vaginal microbiota, measurement of the average concentration of lactic acid in cervicovaginal fluid (CVF) under hypoxic conditions is 1.0 ± 0.2% (*w*/*v*), a concentration associated with a low pH between 3.5 and 4.5 [19] (Table 1). The strong inverse correlation between pH and lactic acid levels indicates that lactic acid is the main acidifier in the lower FRT [19]. Lactic acid has been shown to have antimicrobial activity [32, 33], as well as anti-inflammatory properties on cervicovaginal epithelial cells, which together support a role in lowering the risk of sexually transmitted infection acquisition and transmission [34, 35]. In vitro and at physiological concentrations (55–111 mM) and pH (4.5), lactic acid inactivates 17 different BV-associated bacteria, while not affecting the viability of four vaginal *Lactobacillus* spp. [23]. Under in vitro anaerobic conditions, lactic acid produced by *L. crispatus* and *L. gasseri*, but not H_2_O_2_, was shown to inactivate *Neisseria gonorrhoeae* [36], *Chlamydia trachomatis* [22, 37] as well as *Escherichia coli* [38]. Lactic acid produced by *L. crispatus* inhibits *N. gonorrhoeae* and *Gardnerella vaginalis* as demonstrated in a porcine vaginal mucosa explant model [39].

Interestingly, Antonio et al. found that 94, 95, and 70% of *L. crispatus, L. jensenii* and *L. gasseri* isolates, respectively, but only 9% of *L. iners* strains produce H_2_O_2_ in vitro [40]. Notably, the D-isomer of lactic acid is exclusively produced by *L. crispatus*, *L. jensenii* and *L. gasseri*, and not *L. iners* [41, 42], and while not extensively tested, has been shown to be associated with immobilisation of HIV-1 in mucus [43], as well as being implicated in preventing vaginal bacteria traversing the cervix to initiate upper genital tract infections [41]*.* Thus, the lack of H_2_O_2_-production may represent less beneficial strains of *Lactobacillus* spp. that do not produce high quantities of lactic acid and thus fail to acidify the vagina to low pH [29, 41] or lack the ability to produce D-lactic acid, which appears to be an essential isomer. Further, Tomás et al. [44] found that strains of vaginal *Lactobacillus* species producing H_2_O_2_ in vitro also produced significantly more lactic acid than non-H_2_O_2_-producing strains. Lastly, slow growth or metabolic rates might limit the competitiveness of some *Lactobacillus* spp. leading to lack of dominance when faced with intrinsic (e.g., menses) and extrinsic (e.g., sex) disturbances, thus frequently transitioning to BV-like microbiota comprising a wide array of strict and facultative anaerobes [45] and should also be considered.

## Conclusion

The scientific literature does not support an in vivo antimicrobial role for H_2_O_2_ produced by vaginal *Lactobacillus* spp. The perpetuation of this concept lacks scientific justification. Definitive clarification of the lack of in vivo antimicrobial role for H_2_O_2_ is critical to focus on more plausible mechanisms by which vaginal *Lactobacillus* spp. exert their antimicrobial and beneficial properties, including that of lactic acid. This goal could be achieved by examining, but not limited to, the following:

1. In vitro studies examining the antimicrobial role of *Lactobacillus* spp. metabolites or other factors could consider—and ideally recapitulate—the conditions generally prevailing in vivo, e.g., hypoxia and high antioxidant capacity among others.
2. The direct antimicrobial activity of any factor, whether endogenous products of *Lactobacillus* spp. or exogenous formulation, could be confirmed in the presence of CVF.
3. Epidemiological studies associating vaginal microbiota and reproductive health outcomes could incorporate quantification of putative antimicrobial factors or activities of *Lactobacillus* spp. in ex vivo CVF samples in prospective study designs.

A shift of focus on mechanisms with translational relevance is key for the evidence-based selection of *Lactobacillus* spp. or *Lactobacillus* metabolites, and other factors, in efforts to develop and test novel biotherapeutics for the female reproductive tract.
